# Box–Behnken Design of Experiments of Polycaprolactone Nanoparticles Loaded with Irinotecan Hydrochloride

**DOI:** 10.3390/pharmaceutics15041271

**Published:** 2023-04-18

**Authors:** Basant Salah Mahmoud, Christopher McConville

**Affiliations:** 1 School of Pharmacy, College of Medical and Dental Sciences, University of Birmingham, Birmingham B15 2TT, UK; 2 Hormones Department, Institute of Medical Research and Clinical Studies, National Research Centre, El Buhouth St., Dokki, Cairo 12622, Egypt

**Keywords:** polycaprolactone, irinotecan hydrochloride, nanoparticles, Box–Behnken, design of experiments, yield, size, zeta potential, encapsulation efficiency

## Abstract

Background: The Box–Behnken design of experiments (BBD) is a statistical modelling technique that allows the determination of the significant factors in developing nanoparticles (NPs) using a limited number of runs. It also allows the prediction of the best levels of variables to obtain the desired characteristics (size, charge, and encapsulation efficiency) of the NPs. The aim of this study was to examine the effect of the independent variables (amount of polymer and drug, and surfactant concentration) and their interaction on the characteristics of the irinotecan hydrochloride (IRH)-loaded polycaprolactone (PCL) NPs and to determine the most optimum conditions for producing the desired NPs. Methods: The development of the NPs was carried out by a double emulsion solvent evaporation technique with yield enhancement. The NPs data were fitted in Minitab software to obtain the best fit model. Results: By using BBD, the most optimum conditions for producing the smallest size, highest magnitude of charge, and highest EE% of PCL NPs were predicted to be achieved by using 61.02 mg PCL, 9 mg IRH, and 4.82% PVA, which would yield 203.01 nm, −15.81 mV, and 82.35% EE. Conclusion: The analysis by BBD highlighted that the model was a good fit to the data, confirming the suitability of the design of the experiments.

## 1. Introduction

Polycaprolactone (PCL) is a semi-crystalline and biodegradable polymer with a slow degradation rate, which gives it the advantage of sustained drug release over extended periods of time. Unlike PLGA, PCL degradation does not increase the acidity of the surrounding environment, causing little effect on the homeostasis [1,2]. PCL has permeability to drug molecules including irinotecan hydrochloride (IRH) [3], which is a topoisomerase inhibitor that ceases DNA replication. IRH and its active metabolite (SN38) are not hindered by the multi-drug resistance challenge as they are not recognized by the p-glycoprotein transporter [4]. Therefore, IRH is considered as a favorable chemotherapy which, unlike the majority of chemotherapy agents, does not rely on the O(6)-Methylguanine-DNA methyltransferase (MGMT) methylation mode of action [4].

During the development of nanoparticles (NPs), it is difficult to encapsulate hydrophilic drugs such as IRH into a hydrophobic polymer matrix such as PCL. This is because hydrophilic drugs have a limited affinity for hydrophobic polymers and thus favor the water phase during the emulsification process, which results in low encapsulation efficiency (EE). In addition, the large surface area of the NPs can lead to premature drug release due to a large proportion of the drug being electrostatically attached to the surface, rather than encapsulated within the NPs [3]. Manipulating the different formulation parameters can influence the EE of hydrophilic drugs within the polymer matrix as well as influence the size and charge of the NPs.

The size, PDI, and surface charge of the NPs are critical factors that influence distribution, cellular uptake, membrane interactions, absorption, and stability in vivo [5]. NPs can traverse through the endothelial fenestrations, which vary in size depending on the organ or tumor [6]. A desired size should be less than 500 nm to avoid phagocytosis of the NPs by macrophages [7]. Smaller-sized NPs favor uptake by non-phagocytic cells, leading to an enhanced intracellular drug concentration [8]. However, very small NPs can have minor ligand to receptor interaction [9]. There is no standard size for NPs that can reach and penetrate tumors efficiently. Blood vessels obstruct particles of a large size, while small particles do not reside in tumors for long periods of time [10]. Previous research suggested that 200 nm particles passed through normal blood vessels and were not filtered out in the kidneys but were able to penetrate through leaky blood vessels in the cancer environment by an EPR effect [11].

The charge and hydrophobicity of the NPs can have an impact on the protein corona formed on their surface when administered intravenously. A neutral and hydrophilic surface is more favorable than a hydrophobic surface as it can accumulate plasma proteins [12]. Additionally, a high magnitude of negative net charge is reported to be more favorable than a positive charge due to better accumulation in tumors. On the other hand, positively charged NPs can lead to protein adsorption and adhesion to anionic polyelectrolytes of mucosal layers [13,14].

The design of experiments (DOE) is a valuable statistical tool for developing a correlation between independent key parameters that control the manufacture of NPs. The DOE determines the significant and insignificant factors that contribute towards an output of interest. This facilitates the optimization of key factors using a limited number of runs designed with a statistical foundation [15]. In a DOE, two or more independent variables are employed to develop a factorial design, through which the interactions between variables can be analyzed and the behavior of the formulations can be interpreted [16,17]. NPs with favorable size, charge, and EE can be achieved with a factorial design. The mixture of different variables with different levels could impact the interactions between molecules in the nano-formulations, resulting in different properties [18].

The Box–Behnken design of experiments (BBD) is a response surface method used in DOE. It relies on the combination of low, medium, and high levels of variables. Compared to central composite design, which requires twenty runs and five levels of factors, BBD requires a smaller number of runs including three center points and three levels of variables where it does not consider runs at the extreme levels [19]. No previous BBD studies were conducted for the purpose of optimization of IRH-loaded PCL NPs. Therefore, a BBD was performed in this study using the most optimum formulation parameters developed in our previous study [1] as the center point. This helped us to examine the effect of the independent variables and their interaction on the characteristics of the IRH-loaded PCL NPs, and to determine the most optimum conditions for producing the desired NPs. This was achieved by conducting 15 independent experiments using a solvent evaporation emulsification technique. The size, charge, PDI, and EE were then measured for each formulation and the mean data were fitted into Minitab software to develop the model.

## 2. Materials and Methods

### 2.1. Chemicals

PCL (14,000—50,000 g/mol), polyvinyl alcohol (PVA) (13,000–23,000 g/mol), sucrose, sodium chloride (NaCl), and hydrochloric acid (HCL) were purchased from Sigma-Aldrich (Dorset, Gillingham, UK). IRH was purchased from LGM Pharma (Erlanger, KY, USA). Acetonitrile and dichloromethane (DCM) were purchased from ThermoFisher Scientific (Loughborough, UK).

### 2.2. Double Emulsion (Water in Oil in Water (W/O/W)) Solvent Evaporation Technique

The NP formulations were prepared by dissolving the required amount of IRH in 3 mL dH_2_O containing 10 mg/mL NaCl to form the aqueous phase (W1), and the required amount of amorphized PCL in 10 mL DCM to form the organic phase (O). By using a burette, W1 was added dropwise (60 drops/min) to the organic phase, under gentle stirring, to form the first emulsion, which was subsequently added to 25 mL of the external aqueous phase (W2) containing 25 mL of PVA surfactant and 2.5% NaCl. Following the double emulsification process, sonication was performed on an ice bath (to avoid over-heating by the probe sonicator) for 10 min, followed by solvent evaporation under gentle stirring at room temperature. The NPs were collected by centrifugation at 24,500 rpm (Beckman Coulter Centrifuge, Buckinghamshire, UK) for 30 min.

### 2.3. Enhancement of the Yield of the NPs

Different centrifugation approaches were used to enhance the yield of the NPs. The first approach involved repeated centrifugations (2 times) at 9500 rpm (Beckman Coulter Centrifuge, Buckinghamshire, UK) for 20 min at 25 °C. The second approach involved diluting formulations with dH_2_O, and then centrifugation at 24,500 rpm for 30 min.

(1) $$\%\;Yield=\frac{Mass\;of\;nanoparticles\;obtained}{Mass\;of\;polymer,\;drug\;and\;cryprotectant}\;\times \;100$$

### 2.4. BBD

Fifteen drug-loaded NP formulations were developed by a double emulsion modified method, as described previously. Table 1 presents the coded levels of the independent variables in BBD. Table 2 provides the actual levels corresponding to each of the codes in BBD.

### 2.5. Collection and Lyophilization of NPs

The NP formulations were collected by centrifugation at 24,500 rpm (Beckman Coulter Centrifuge, Buckinghamshire, UK). The NPs were then washed twice with dH_2_O to remove the free drug, frozen overnight at −80 °C in 5% sucrose solution and subsequently lyophilized at 0.01 mbar and −85 °C for 48 h using Labconco lyophilizer (Kansas city, MI, USA).

### 2.6. Measurement of Particle Size and Zeta Potential

In total, 2 mg (n = 3) of the lyophilized NPs were dispersed in 3 mL of dH_2_O and analyzed for their hydrodynamic diameter, PDI, and zeta potential using a dynamic light scattering (DLS) Zetasizer (Malvern, Worcestershire, UK).

### 2.7. Measurement of EE by HPLC

The analysis of IRH was conducted by using Agilent HPLC (Agilent Technologies 1260 infinity II) with a quaternary gradient pump. A C18 column (150 mm × 4.6 cm) with 5 μm particle size was used to perform the separation at 25 °C (ThermoFisher Scientific, Loughborough, UK).

#### Detection of IRH by Ion Pair Method

An injection volume of 20 μL, a run time of 10 min, and a flow rate of 1.00 mL/min were selected for the analysis. The mobile phase was composed of an ion pair solution of 1.2 g octane-l-sulfonic acid in 500 mL dH_2_O (solution A), and 13.6 g potassium dihydrogen phosphate dissolved in 500 mL dH_2_O (solution B). In addition to solutions A and B, acetonitrile was added to obtain a ratio of 30:30:40 *v*/*v*/*v* at pH 3, using orthophosphoric acid. The analysis was performed with a UV detector at 265 nm wavelength.

The un-encapsulated drug (free drug) was determined in the supernatant of each NP formulation via HPLC analysis. EE was calculated using the following equation:

(2) $$\mathrm{EE}\;(\%)=\frac{\mathrm{Initial}\;\mathrm{drug}\;\mathrm{amount}-\mathrm{Drug}\;\mathrm{in}\;\mathrm{the}\;\mathrm{supernatant}}{\mathrm{Initial}\;\mathrm{drug}\;\mathrm{amount}}\times 100$$

### 2.8. Statistical Analysis

Data produced from BBD were fitted using the Minitab software version 19.2020.2.0 to obtain the best-fit model represented by the R^2^. Analysis of variance (ANOVA) was performed to ensure the model was a good fit. F-tests and P-values were employed to determine the significance of the regression coefficient. Data fitted to the software were the mean of 3 replicated measurements. A Pareto chart was used to determine the significant variables. Finally, 2D and 3D plots were used to provide a graphical representation of the model.

## 3. Results

### 3.1. Yield of NPs

#### Characterization of NPs before and after Yield Enhancement

**Figure 1:**
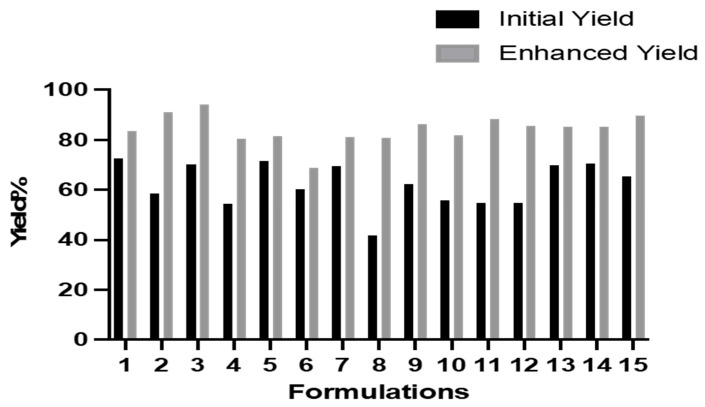
Initial and enhanced yield percentage of NP formulations.

As shown in Table 3 and Table 4, the increased centrifugation speed and reduced viscosity, by using dH_2_O for dilution, resulted in a significant impact on both the size and PDI of the NPs, while the zeta potential of the NPs became significantly higher in magnitude, indicating more stability and less agglomeration. There was a varying impact on the EE, with most formulations showing no impact of the increased centrifugation. Figure 1 demonstrates that the yield of each formulation was increased with the higher centrifugation speed compared to the repeated centrifugation at a lower speed.

### 3.2. BBD Predicted Model and Best Fit Regression Coefficient for Size

The size (Y1) (dependent variable) obtained at three different independent variables (A, B and C), respectively, as presented in the model, was subjected to multiple regression analysis to fit the response with the experimental data and to produce a second-order polynomial model as provided by Equation (3).

(3) Size=235.7+15.23 A −18.76 B −29.24 C +12.4 A∗A +11.2 B∗B  +52.5 C∗C −1.5 A∗B +53.4 A∗C −0.2 B∗C

β0 represents the intercept with the y-axis, while β1 to β9 represent the regression coefficients for the linear, square, and interaction effects, as shown in Table 5. The value for R^2^ of equation 3 was found to be 0.94, which indicated a good correlation between the experimental and predicted data. R^2^ values greater than 0.50 were considered reasonable, as reported previously [20]. The adjusted R^2^ was 0.83 which implied that 17.00% of the data were not explained by the model. A non-significant lack of fit (>0.05) indicated that the model is significant for the response. ANOVA was used to test the significance of the model, and it presented a significant model for describing the size of the NPs expressed in Prob > F value of 0.013. A probability of 0.05 or less is regarded as a significant effect of the independent factors on the response.

The size of the different formulated NPs ranged from 203.60 ± 1.04 to 388.10 ± 6.45. The model implies that the size of the NPs is significantly affected by the amount of drug and surfactant concentration, which have an antagonistic effect on the response. However, the concentration of the surfactant square and the interaction between the polymer and surfactant were shown to have a significant synergistic effect on the size of the NPs, as shown in Table 5. The main effects of A, B, and C represent the average results for changing one variable at a time from its low to high level. The interaction terms AB, AC, and BC represent the change in response when two variables are changed simultaneously. The positive coefficients indicate the synergistic effect, and the negative coefficients indicate an antagonistic effect on the response. The significant values for all contributing factors are provided in Table 5. The theoretical and experimental values are presented in Table 6. Table 7 provides the statistical analysis for the size of the NPs.

**Figure 2:**
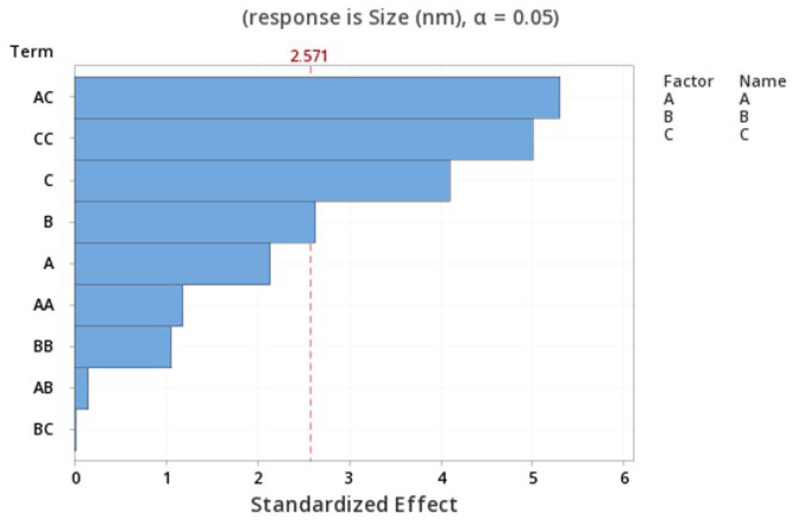
Pareto chart expressing the standardized effect of independent variables and their interaction on the size of the NPs.

The standardized effect of the independent variables and their interaction on the response is presented via the Pareto chart (Figure 2), which ranks the effect of the variables on the output in terms of its significance. The Pareto chart shows that the AC, C^2^, and C have the most significant effect on the size of the NPs (*p* = 0.003).

**Figure 3:**
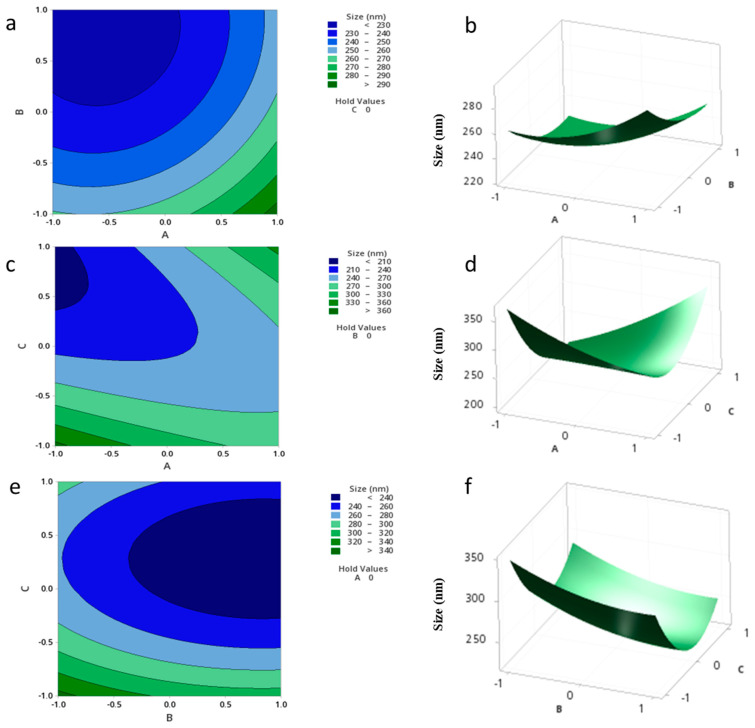
Contour plots (**a**,**c**,**e**) and Surface plots (**b**,**d**,**f**) showing the effect of variables (A–C) on the size of the NPs.

The relationship between the independent and dependent variables was further highlighted by plotting 2D contour and 3D surface plots. The effects of two variables and their interaction on the size of the NPs are expressed in Figure 3 at a fixed level of the third variable. It was determined from the model that the correlation between A and C has a significant effect on the size of the NPs. A smaller NP size ranging from 203.60 to 210.00 nm could be obtained with an A range of −1 to −0.7 and a C range from 1 to 0.4 (Figure 3c,d). It is evident from the contour and surface plots that increasing the level of C while reducing the level of A results in a reduction in the size of the NPs. On the contrary, the relationships between A and B, and B and C were non-significant, as shown by the circular contour plots and the saddle 3D plots (Figure 3a,b,e,f).

The highest significant effect was shown to be the interaction between the polymer and surfactant, with a contribution of 34.04%. The contribution percentage was calculated according to Equation (4) [21].

(4) $$\mathrm{Contribution}\%=\frac{\mathrm{SSA}}{\mathrm{SS}}\;\times \;100$$

The SSA refers to the sum of squares for each individual factor, whereas SS refers to the sum of squares of the model.

### 3.3. BBD Predicted Model and Best Fit Regression Coefficient for Zeta Potential

The zeta potential (Y2) (dependent variable) obtained at three different independent variables (A, B, and C) was subjected to multiple regression analysis to fit the response with the experimental data and produce a model of a second-order polynomial equation as follows:

Zeta Potential (mV) = −15.60 + 1.277 A + 1.754 B − 1.460 C + 1.94 A ∗ A − 2.18 B ∗ B + 2.59 C ∗ C(5)

The value for R^2^ of equation 5 was found to be 0.78 and 0.61 for the adjusted R^2^ with a non-significant lack of fit (0.06). The ANOVA represents a significant model for describing the charge of the NPs expressed in Prob > F value of 0.023.

The zeta potential of the NPs for the different formulations ranged from −7.63 ± 0.25 to −19.63 ± 2.56 mV. The model demonstrates that the charge of the NPs is significantly impacted by the amount of drug and the squared concentration of the surfactant. This significant effect is shown to be a synergistic effect, as shown in Table 8. The significant values for all contributing factors are provided in Table 8. The theoretical and experimental values are presented in Table 9. Table 10 provides the statistical analysis for the charge of the NPs.

**Figure 4:**
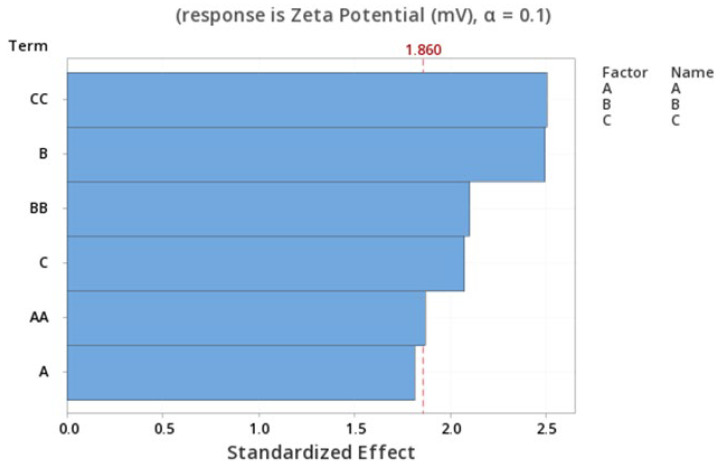
Pareto chart expressing the standardized effect of independent variables and their interaction on the charge of the NPs.

The standardized effect of the independent variables and their interaction on the response are presented via the Pareto chart (Figure 4), which ranks the effect of the variables on the outcome by significance. As presented in the figure, the only significant factors are C^2^ and B (*p* = 0.03).

**Figure 5:**
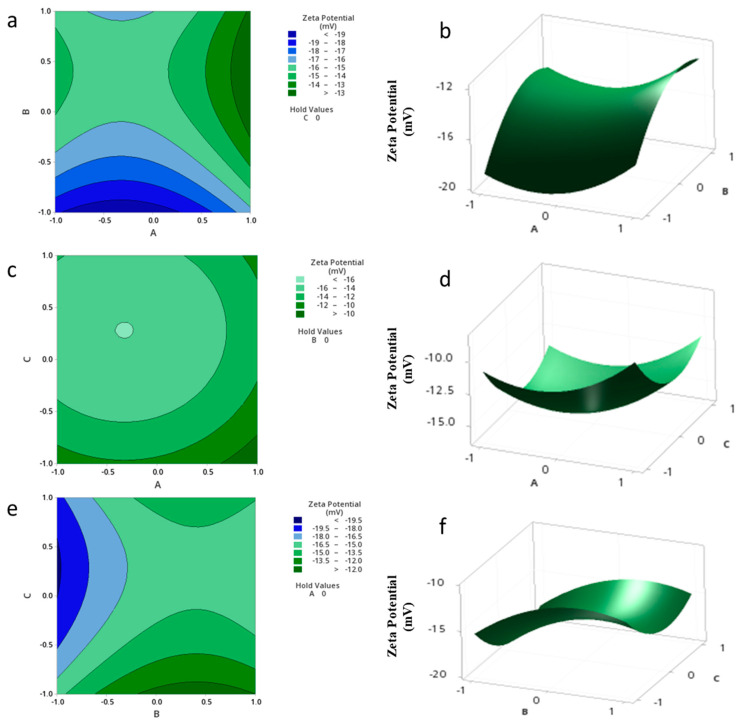
Contour plots (**a**,**c**,**e**) and Surface plots (**b**,**d**,**f**) showing the effect of variables (A–C) on the charge of the NPs.

The relationship between the independent and dependent variables was further highlighted by plotting surface and contour plots, as expressed in Figure 5. As shown in the figures, there were no significant interactions between the independent parameters as expressed via the contour plots, and quadratic (Figure 5B,F) and saddle surface plots (Figure 5D).

The highest significant effect was shown to be the squared concentration of the surfactant (C^2^) with 24.84% contribution.

### 3.4. BBD Predicted Model and Best Fit Regression Coefficient for EE%

The EE% (Y3) (dependent variable) obtained at three different independent variables (A, B, and C) was subjected to multiple regression analysis to fit the response with the experimental data and produce a model of a second-order polynomial equation.

EE% = 64.40 − 2.914 A + 9.547 B − 2.919 C + 5.82 A ∗ A + 2.12 B ∗ B + 2.50 C ∗ C + 1.19 A ∗ B + 1.13 A ∗ C + 3.59 B ∗ C(6)

The value of R^2^ for equation 6 was found to be 0.97, and the adjusted R^2^ was 0.94 with a non-significant lack of fit (0.08). The ANOVA represents a significant model for describing the EE% of the NPs expressed in Prob > F value of 0.001.

The EE% of the NPs for the different formulations ranged from 52.44% ± 0.10 to 82.42% ± 0.03. As shown in Table 11, the model shows that the EE% of the NPs is significantly influenced by the amount of drug, the interaction between the drug and surfactant, and the quadratic amount of polymer, which all have a synergistic effect on EE%. Whereas, the polymer amount and concentration of surfactant have antagonistic significant effects on the EE%. The theoretical and experimental values are provided in Table 12. Table 13 provides the statistical analysis for the EE% of the NPs.

**Figure 6:**
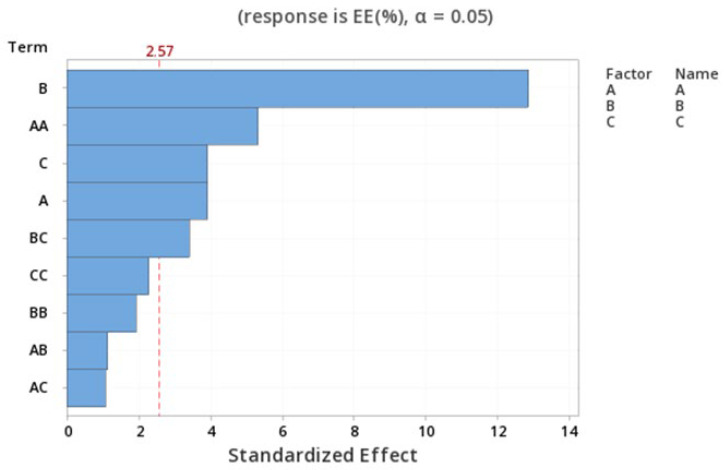
Pareto chart expressing the standardized effect of independent variables and their interaction on the EE% of the NPs.

The standardized effect of the independent variables and their interaction on the response are presented via the Pareto chart (Figure 6), which shows that B, A^2^, and C are the most significant factors (*p* = 0.000, 0.003 and 0.011, respectively).

**Figure 7:**
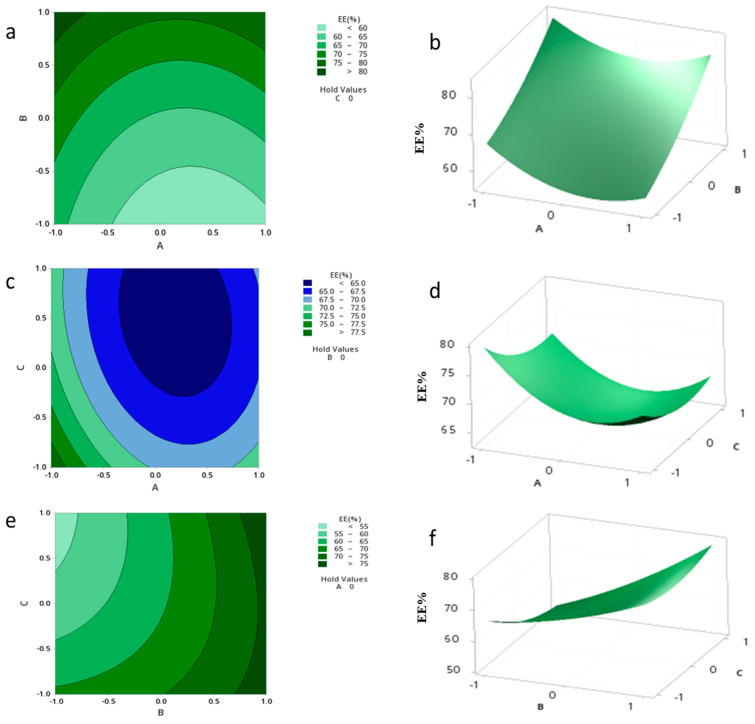
Contour plots (**a**,**c**,**e**) and Surface plots (**b**,**d**,**f**) showing the effect of variables (A–C) on the EE% of the NPs.

The relationship between the independent and dependent variables was further highlighted by plotting surface and contour plots. It was determined from the contour and surface plots (Figure 7e,f) that an EE of 70.00% or higher could be obtained with a B range from −1 to 1 level and a C range from −0.9 to 1 level. The highest significant effect was shown to be the amount of drug, with the highest percent of contribution of 67.71%.

## 4. Discussion

The W/O/W Solvent Evaporation Technique was used to develop IRH-loaded PCL NPs with the addition of NaCl to help maintain the osmotic pressure of the inner and outer phases, which reduces the partitioning of the water soluble IRH into the external aqueous phase, hence enhancing its encapsulation.

The yield of the NPs was enhanced by reducing the viscosity of the solution by diluting the formulation with dH_2_O before centrifugation. This has also impacted the charge of the NPs by increasing the magnitude of the negative zeta potential, which maintains the stability of the NPs by avoiding their agglomeration. A high magnitude of the negative charge produced via high-speed centrifugation indicated higher stability of the NPs compared to the repeated centrifugation approach. It was evident that the repeated spins have compromised the stability of the NPs represented in a lower value of the negative zeta potential, as expressed in Table 3. The formulations with the enhanced yield were then used in the BBD model.

The purpose of using BBD was to examine the effect of different formulation variables on the size, charge, and EE of the NPs by changing the levels of the formulation variables from low to high. The goal was to reduce the size, increase the magnitude of the negative charge and maximize the EE% of the NPs. It was found that a size ranging from 203.60 ± 1.04 to 388.10 ± 6.45 nm, a zeta potential ranging from −7.63 ± 0.25 to −19.63 ± 2.56 mV, and EE% ranging from 52.44% ± 0.10 to 82.42% ± 0.03 were obtained via the double emulsion solvent evaporation techniques using different amounts of polymer (54, 108 and 162 mg), drug (3, 6 and 9 mg), and surfactant concentrations (2, 4 and 6%).

The BBD was conducted using 15 independent runs in random order at three different levels: low, medium, and high. The polynomial equations, contour plots, and 3D plots were used to determine the relationship between the independent variables and response. Multiple regression and ANOVA analyses were performed for the fitted polynomial model. The model was found to be a significant fit for size, charge, and EE% responses. The 2D contour and 3D surface plots provided a graphical view of the dependent and independent variables. Contour plots with an elliptical shape indicate that the interaction between the independent variables is significant, whereas circular contour plots indicate a non-significant relationship [22,23]. Through contour plots, optimum levels of the response could be easily detected.

The smallest size of the NPs was 203.60 ± 1.04, which was achieved by using −1, 0, and 1 levels equivalent to 54 mg, 6 mg, and 6% of the polymer, drug, and surfactant, respectively. An average PDI of 0.18 to 0.49 substantiates a narrow size distribution. It was previously reported that NPs up to 200 nm size were monodispersed, showed higher stability, and successfully crossed biological barriers. However, a larger size could allow for higher entrapment of the drug and could enhance their efficacy [24,25].

The model suggests that the high amount of drug resulted in a significant size reduction. This implies that the high amount of drug was required to interact with the polymer and avoid agglomeration caused by excessive drug concentration, which aided in reducing the size [26]. The concentration of the surfactant had a significantly antagonistic effect on the size, but when the concentration was squared, it had an opposite effect, leading to a larger particle size. Similar results were previously reported in which a high concentration of the surfactant increased the size of the NPs due to increased viscosity [27]. A high stability is achieved in the presence of an adequate level of the surfactant, which when increased beyond that level can lead to adverse effects on the size of the NPs [27]. Similarly, the interaction between the polymer and surfactant was shown to increase the size of the NPs, which is suggested to be due to increased hydrophobicity [28]

The highest value of negative charge achieved in this model was −19.63 ± 2.56 mV by using 0, −1, and 1 levels equivalent to 108 mg, 3 mg, and 6% of the polymer, drug, and surfactant, respectively. The negative charge of the NPs could be due to the carbonyl groups of the PCL, which create a negative charge on the surface of the NPs. The shift in the values of the charge could be due to the different concentrations of the PCL as well as the shielding effect of the PVA because of its interaction with the PCL or the entrapped drug [29]. The negative charge of the NPs could also be due to the interaction of the polymer with the hydrophobic region of the surfactant, whereas the hydrophilic region of the surfactant stays in the aqueous phase. This interaction creates steric and electric stabilization [30]. The model suggests that the amount of drug and squared surfactant concentration have a significant impact on the charge of the NPs. This could be a result of the high viscosity imposed by the high surfactant concentration in addition to the positively charged drug, which could lead to a reduction in the magnitude of the charge of the NPs. IRH is reported to have a more positive charge in its lactone form than its carboxylate form [31].

The stability of the NPs is achieved by maintaining a high value of the negative zeta potential, indicating a high level of repulsion between particles which is necessary to prevent aggregation of the particles [32]. There was no significant difference between the neutral and negatively charged NPs in circulation half-life, as previously demonstrated [33]. On the contrary, increasing the positive charge on PEGylated liposomes reduced their residence in the blood and enhanced their uptake by the liver in a previous study [34]. Moreover, the positively charged NPs were found to have an enhanced interaction with the negatively charged cell membrane of the macrophages and phagocytosis compared to the negatively charged or neutral NPs [35].

The highest EE% in this BBD was found to be 82.42% ± 0.03, achieved with −1, 1, and 0 equivalent to 54 mg, 9 mg, and 4% of the polymer, drug, and surfactant, respectively. A low level of PCL and a high level of drug achieved the highest EE, which confirms that the amount of PCL was sufficient to entrap the drug and avoid agglomeration. PCL was reported to efficiently entrap drugs such as tamoxifen, dapivirine, and docetaxel, which was dependent on the solubility of the drugs within the polymer [36,37,38,39]. The model implies that EE% of the NPs is significantly influenced by the amount of drug, which is similarly proven in other research, where the concentration of cisplatin was directly proportional with EE% in PLGA-mPEG NPs [40]. On the other hand, our results exhibited a significant decrease in EE% upon increasing the amount of polymer. However, the amount of polymer squared had a synergistic effect on EE%. The interaction between the drug and surfactant helped to keep the drug entrapped in the NPs. However, with high levels of surfactant, the EE% was negatively affected. This is possibly due to the formation of hydrogen bonds between the hydroxyl groups of the PVA molecules, leading to an increased size and decreased EE% [41].

A theoretical optimum condition could be achieved by setting the desired characteristics of a minimum particle size and charge and a maximum EE%, using the desirability function in the Minitab software. The optimum condition, with a desirability of 0.88, was predicted to be achieved by using −0.87 level of A, 1 level of B, and 0.41 level of C, which would produce NPs with a size of 203.01 nm, a charge of −15.81 mV, and an EE% of 82.35%. The relationship between the coded and actual values was provided according to the following Equation:

(7) $$x_{i}=\frac{X_{i}-x_{0}}{\Delta X_{i}}\;\;\;\;\;\;i=1,2,3,\;\ldots .,\;k$$

where *x_i_* is the coded value of an independent variable; *X_i_* is the actual value of an independent variable; *X*_0_ is the actual value of an independent variable at the center point; and Δ*X_i_* is the step change [42]. According to Equations (8)–(10), the relationship between the coded and actual values of the independent variables of the amount of polymer and drug, and surfactant concentration, respectively, was calculated as follows:

(8) $$-0.87=\frac{X_{1}-108}{54}\;\;\;\;\;\;\;\;X_{1}=61.02\;\mathrm{mg}$$

(9) $$1=\frac{X_{2}-6}{3}\;\;\;\;\;\;\;\;\;\;\;\;X_{2}=9\;\mathrm{mg}$$

(10) $$0.41=\frac{X_{3}-4}{2}\;\;\;\;\;\;\;\;\;X_{3}=4.82\%$$

## 5. Conclusions

The development of NPs with optimum properties is crucial for their success in the clinic. Therefore, BBD was employed in that regard to allow the investigation and selection of optimum values of the independent factors, resulting in the smallest size, highest magnitude of surface charge, and highest EE%, which were found to be achieved by using 61.02 mg of PCL, 9 mg of drug, and 4.82% of surfactant. This study highlighted that the size of the IRH-loaded PCL NPs was mostly impacted by the interaction between the PCL and PVA, and that the charge of the NPs was mostly influenced by the squared concentration of the PVA. Furthermore, the highest influencing factor towards the EE% was the amount of drug.

## Figures and Tables

**Table 1:** Box–Behnken coded and actual values.

Variable	Coded Values		
	−1 (Low)	0 (Medium)	1 (High)
A = PCL Amount (mg)	54	108	162
B = IRH Amount (mg)	3	6	9
C = PVA Concentration (%)	2	4	6
Y1 = Size	-	-	-
Y2 = Zeta Potential	-	-	-
Y3 = EE	-	-	-

**Table 2:** Box–Behnken 3-level factorial design with the actual values.

Experiment	Variable 1 (A; mg)	Variable 2 (B; mg)	Variable 3 (C; %)
1	54	3	4
2	162	3	4
3	54	9	4
4	162	9	4
5	54	6	2
6	162	6	2
7	54	6	6
8	162	6	6
9	108	3	2
10	108	9	2
11	108	3	6
12	108	9	6
13	108	6	4
14	108	6	4
15	108	6	4

**Table 3:** Mean size, PDI, zeta potential, and EE% of NPs before yield enhancement.

Formulation	Size (nm)	PDI	Zeta Potential (−mV)	EE%
F1	281.63	0.30	−3.48	61.46
F2	288.83	0.38	−3.68	73.02
F3	708.93	0.52	−3.00	89.88
F4	268.43	0.33	−3.02	70.28
F5	373.43	0.48	−4.78	80.32
F6	476.70	0.55	−4.88	74.65
F7	378.47	0.18	−3.80	76.90
F8	250.37	0.43	−3.28	65.31
F9	294.03	0.44	−4.68	67.24
F10	520.80	0.55	−4.55	75.30
F11	351.20	0.51	−3.16	64.15
F12	1552.00	1.00	−2.69	84.11
F13	314.33	0.25	−3.32	74.60
F14	339.70	0.32	−3.593	70.89
F15	368.97	0.32	−3.09	67.83

**Table 4:** Mean size, PDI, zeta potential, EE%, and IRH loading% of NPs after yield enhancement. * indicates significance, while the arrow indicates either an increase (↑) or decrease (↓) compared to the data in Table 3.

Formulation	Size (nm)	PDI	Zeta Potential (−mV)	EE%	IRH Loading%
F1	244.40	0.31	−17.60 *↑	65.25	0.55
F2	298.80	0.33	−19.23 *↑	59.86 *↓	0.35
F3	222.80 *↓	0.30 *↓	−14.36 *↑	82.42	1.84
F4	271.10	0.22 *↓	−12.16 *↑	81.81 *↑	1.62
F5	388.10	0.49	−13.10 *↑	80.34	1.60
F6	290.80 *↓	0.27 *↓	−7.63 *↑	69.42	0.81
F7	203.60 *↓	0.42 *↑	−13.86 *↑	73.74	0.96
F8	320.00 *↑	0.18 *↓	−9.68 *↑	67.35	0.67
F9	344.00 *↑	0.30 *↓	−12.60 *↑	66.95	0.51
F10	294.00 *↓	0.32 *↓	−13.33 *↑	78.41	1.85
F11	305.10 *↓	0.41 *↓	−19.63 *↑	52.44 *↓	0.39
F12	254.30 *↓	0.34 *↓	−15.16 *↑	78.24 *↓	1.77
F13	237.26 *↓	0.30	−16.06 *↑	64.63 *↓	0.74
F14	241.70 *↓	0.39	−14.96 *↑	63.53 *↓	0.73
F15	228.13 *↓	0.37	−15.76 *↑	65.03	0.71

**Table 5:** Summary of regression coefficient analysis for the response Y1 (Size).

Coefficient	β0	β1 (A)	β2 (B)	β3 (C)	β4 (A ∗ A)	β5 (B ∗ B)	β6 (C ∗ C)	β7 (A ∗ B)	β8 (A ∗ C)	β9 (B ∗ C)
Size (nm)	235.70	15.23	−18.76	−29.24	12.4	11.2	52.5	−1.5	53.4	−0.2
*p* value	0.000	0.086	0.047	0.009	0.290	0.337	0.004	0.886	0.003	0.985

**Table 6:** Experimental and theoretical values with residuals of the response Y1.

Formulation No.	Experimental (Observed) Value of Size	Theoretical (Predicted) Value of Size	Residuals	%Error
1	244.40	261.29	−16.89	−6.91
2	298.80	294.79	4.01	1.34
3	222.80	226.81	−4.01	−1.80
4	271.10	254.21	16.89	6.23
5	388.10	368.06	20.04	5.16
6	290.80	291.66	−0.86	−0.30
7	203.60	202.74	0.86	0.42
8	320.00	340.04	−20.04	−6.26
9	344.00	347.15	−3.15	−0.92
10	294.00	310.03	−16.03	−5.45
11	305.10	289.07	16.03	5.25
12	254.30	251.15	3.15	1.24
13	237.26	235.70	1.56	0.66
14	241.70	235.70	6.00	2.48
15	228.13	235.70	−7.57	−3.32

**Table 7:** ANOVA, degree of freedom (DF), sum of squares (SS), mean of squares (MS), and Fischer’s ratio (F-value) for the size of the NPs.

Regression	DF	SS	MS	F Value	*p* Value
Size (nm)	9	33,533.00	3725.90	9.15	0.01

**Table 8:** Summary of regression coefficient analysis for the response Y2 (Zeta potential).

Coefficient	β0	β1	β2	β3	β4	β5	β6
Zeta Potential (mV)	−15.60	1.27	1.75	−1.46	1.94	−2.18	2.59
*p* value	0.000	0.107	0.038	0.072	0.099	0.069	0.037

**Table 9:** Experimental and theoretical values with residuals of the response Y2.

Formulation No.	Experimental (Observed) Value of Charge	Theoretical (Predicted) Value of Charge	Residuals	%Error
1	−17.60	−17.91	0.31	−1.76
2	−19.23	−17.27	−1.95	10.14
3	−14.36	−16.32	1.95	−13.57
4	−12.16	−11.85	−0.31	2.54
5	−13.10	−11.20	−1.89	14.42
6	−7.63	−8.01	0.37	−4.84
7	−13.86	−13.48	−0.37	2.66
8	−9.68	−11.57	1.89	−19.52
9	−12.60	−14.17	1.57	−12.46
10	−13.33	−13.26	−0.06	0.45
11	−19.63	−19.69	0.06	−0.30
12	−15.16	−13.58	−1.57	10.35
13	−16.06	−15.60	−0.46	2.86
14	−14.96	−15.60	0.063	−4.21
15	−15.76	−15.60	−0.16	1.01

**Table 10:** ANOVA, DF, SS, MS, and F-Value for the charge of the NPs.

Regression	Df	SS	MS	F Value	*p* Value
Charge	6	114.08	19.01	4.79	0.02

**Table 11:** Summary of regression coefficient analysis for the response Y3 (EE%).

Coefficient	β_0_	β_1_	β_2_	β_3_	β_4_	β_5_	β_6_	β_7_	β_8_	β_9_
EE%	64.39	−2.91	9.54	−2.91	5.82	2.11	2.49	1.19	1.13	3.58
*p* value	0.000	0.011	0.000	0.011	0.003	0.111	0.072	0.308	0.331	0.019

**Table 12:** Experimental and theoretical values with residuals of the response Y3.

Formulation No.	Experimental (Observed) Value of EE%	Theoretical (Predicted) Value of EE%	Residuals	%Error
1	65.25	66.90	−1.65	−2.52
2	59.86	58.68	1.18	1.97
3	82.42	83.60	−1.18	−1.43
4	81.81	80.16	1.65	2.01
5	80.34	79.68	0.66	0.82
6	69.42	71.59	−2.17	−3.12
7	73.74	71.58	2.16	2.94
8	67.35	68.01	−0.66	−0.98
9	66.95	65.97	0.98	1.47
10	78.41	77.89	0.52	0.66
11	52.44	52.96	−0.52	−0.99
12	78.24	79.22	−0.98	−1.26
13	64.63	64.40	0.23	0.36
14	63.53	64.40	−0.87	−1.36
15	65.03	64.40	0.63	0.97

**Table 13:** ANOVA, DF, SS, MS, and F-Value for the EE% of the NPs.

Regression	Df	SS	MS	F Value	*p* Value
EE%	9	1076.89	119.65	27.02	0.001

## Data Availability

The datasets and materials used and analyzed during the current study are available from the corresponding author upon reasonable request.
